# Long-Term Follow-up After Ovariectomy Reveals Correlations Between Bone Marrow Adiposity and Trabecular Bone Quality in the Proximal Metaphysis of Tibiae in Rats

**DOI:** 10.1007/s00223-024-01298-x

**Published:** 2024-10-07

**Authors:** Maxime Bedez, Guillaume Falgayrac, Hélène Béhal, Émeline Cailliau, Jérôme Delattre, Xavier Coutel, Cécile Olejnik

**Affiliations:** 1 MABLab – Marrow Adiposity & Bone Laboratory, Faculté de Chirurgie Dentaire de Lille, Univ. Lille, Lille, CHU Lille, Univ. Littoral Côte d’Opale, ULR 4490, Pl. de Verdun, Lille, France; 2 Biostatistics Department, CHU Lille, 59000 Lille, France

**Keywords:** Bone marrow adiposity, Bone, Bone microarchitecture, µCT, Bone mineral density distribution, Bone ultrastructure, Scanning electron microscopy, Raman microspectroscopy, Rat model

## Abstract

This study aimed to evaluate the correlation between BMAT and bone quality, describe the long-term effects of ovariectomy on bone, and investigate BMAT's spatial distribution. Fifteen-months-old female Sprague‒Dawley rats were studied, comparing ovariectomized (OVX, *n* = 22) and sham-operated (SHAM, *n* = 11) groups at 6 months. Tibias were analyzed for bone microarchitecture, BMAT (microcomputed tomography), mineral parameters (quantitative backscattered electron imaging), and bone composition (Raman microspectroscopy). The OVX tibias showed severe trabecular bone loss (lower bone volume/total volume, *p* < 0.001) with increased BMAT (higher adipose volume per marrow volume, *p* < 0.001), decreased mineral content (lower calcium concentration, *p* < 0.001), and altered organic components (lower mineral/matrix ratio in new bone, *p* = 0.03 trabecular surface, *p* < 0.001 trabecular core). When the data are pooled over both groups (SHAM and OVX), the adipose volume/marrow volume ratio was negatively correlated with bone volume/total volume (*r* =  − 0.79, *p* < 0.001) and mineral/matrix ratio (*r* =  − 0.37, *p* = 0.04 trabecular surface; *r* =  − 0.65, *p* < 0.001 trabecular core) and positively correlated with crystallinity (*r* = 0.55, *p* = 0.001 trabecular surface; *r* = 0.49, *p* = 0.006 trabecular core). The mineral/matrix ratio of trabecular surface new bone was strongly negatively correlated with the adipose compartment nearest to the bone surface. These findings suggest mechanisms underlying BMAT's role in bone resorption.

## Introduction

Osteoporosis is marked by an imbalance in bone remodeling due to estrogen deficiency during menopause, leading to changes in bone quality [1]. Fracture risk depends on both bone quantity and quality. Bone loss mainly affects trabecular bone and increases bone marrow adipose tissue (BMAT) volume in specific skeletal sites. There is a well-established negative correlation between BMAT and bone quality in settings of osteoporosis [2] that has historically been explained by two hypotheses: a shift in progenitor differentiation favoring adipocytes over osteoblasts or adipocytes regulating the microenvironment to promote bone resorption [3]. As correlation does not necessarily equate to causation, emerging literature also reveals that bone loss in settings of ovariectomy, diabetes, and with age can occur independent of BMAT expansion [4–6]. This remains an important point to clarify as inhibiting fatty acids in vitro prevents adipocyte lipotoxicity in osteoblasts [7]. Previous research also indicates that BMAT in the proximal tibia is closer to trabecular bone than in the mandible, clustering even closer during osteoporosis like conditions [8, 9]. Our hypothesis is that BMAT undergoes quantitative expansion and spatial distribution changes in osteoporosis.

Osteoporotic bone shows lower calcium content and specific bone mineralization profiles [10]. Environmental scanning electron microscopy (ESEM) can differentiate between newly formed and older, mature bone based on calcium content [11]. Bone strength is affected by bone quantity, ultrastructural composition, and calcium density distribution [12]. Raman microspectroscopy precisely analyzes mineral and organic components, distinguishing new from old bone [13, 14].

This study explores both normal and osteoporotic bone through relative tissue age extrapolated by equivalent calcium content and BMAT spatial distribution, an area previously understudied. We assessed the role of BMAT distribution in bone microarchitecture, mineral parameters, and composition in new and old bone. Using a surgical model of osteoporosis induced by ovariectomy in female rats, we aimed to evaluate the relationship between BMAT and bone quality under both normal and osteoporosis conditions. Secondary objectives included examining the long-term effects of ovariectomy on bone microarchitecture, mineral parameters, and composition, and investigating these parameters across three distinct BMAT spatial compartments.

## Material and Methods

### Experimental Rat Model and Sample Preparation

The guidelines for reporting animal research were followed (ARRIVE 2.0). The protocol (APAFIS#4197–2,015,091,514,435,707) was approved by the National Committee on Ethics in Animal Experimentation (CEEA 075) and legal requirements in France for the care and use of animals were followed. Thirty-three Sprague‒Dawley rats (*Rattus norvegicus*) aged 6 months and with homogeneous weights at baseline (Janvier Lab, Laval, France) [8] were studied. The rats were housed at the animal facility at the University of Lille (DHURE). The rats (*n* = 3 per cage) were housed in type 4 cages filled with Lignocel™ bedding and provided with horizontal tubes for climbing under controlled conditions at 22 ± 2 °C on a 12-h light/12-h dark cycle. Each animal was monitored daily and observed to determine any signs of poor adaptation to its environment. Assignments and surgeries were performed at 6 months by the provider to constitute a sham surgery group (SHAM, *n* = 11) and an ovariectomy group (OVX, *n* = 22). The assignment of the rats to groups was random. All experimenters were aware of the group allocation during performance of the experiment, result evaluation, and data analysis. The rats were sacrificed by exsanguination under anesthesia at 15 months of age (9 months post-OVX).

We previously performed and published our method for the collection and preparation of the tibia samples [8]. The right tibias were subjected to analysis of the bone microarchitecture using microcomputed tomography (µCT). The tibias were harvested, fixed for 48 h in 10% neutral buffered formalin (NBF), and then stored in phosphate-buffered saline (PBS) for a first µCT acquisition. Subsequently, they were prepared for analysis of BMAT via decalcification, osmium tetroxide staining, and storage in PBS, for a second µCT acquisition. The left tibias were used for the analysis of mineral parameters through quantitative backscattered electron imaging (qBEI) and for the analysis of bone composition through Raman microspectroscopy. The left tibias were fixed in 70% ethanol for 48 h before being embedded in polymethylmethacrylate (PMMA) resin. They were then sectioned and polished to a thickness of 100 µm.

### X-Ray Microcomputed Tomography

The data and analysis protocol for bone microarchitecture and BMAT content investigation were previously published by our laboratory team [8]. The initial acquisitions were performed using a Skyscan 1172 µCT device (Bruker MicroCT, Kontich, Belgium) with the following parameters: isotropic voxels of 10 µm^3^, 80 kVp, 100 µA, an Al−Cu filter, an integration time of 2400 ms, and a rotation step of 0.5° over 180°. The data acquisition, reconstruction, analysis, and three-dimensional visualization were conducted using the following software: Nrecon™, Dataviewer™, CTAn™, and CTVox™ (Bruker µCT, Kontich, Belgium). Each bone sample was assessed before and after decalcification and osmium tetroxide staining. Osmium tetroxide binds to lipids stored in the cytoplasm of bone marrow adipocytes (BMAds), revealing BMAT in µCT. The region of interest is a cross-sectional slice of 2 mm thickness, located 1.5 mm below the growth plate. This protocol was carried out on the 33 tibias in this study. Bone density was represented by the ratio of bone volume to total volume (BV/TV, expressed as a percentage). BMAT was represented by the ratio of adipose volume to marrow volume (AdV/MaV, expressed as a percentage). The bone marrow was divided into 20 µm compartments relative to the surface of the trabecular bone (D1: 0–20 µm, D2: 20–40 µm, D3: 40–60 µm) to study the spatial distribution of BMAT.

### Environmental Scanning Electron Microscopy and Bone Mineral Density Distribution

**Fig. 1:**
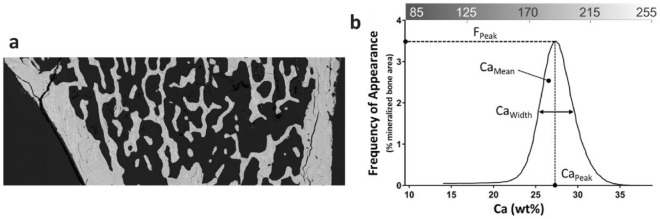
Grayscale BSE image of the trabecular and cortical bone in a left tibia from the SHAM group (**a**). The grayscale curve ranging from 85 to 255 (upper *x*-axis), with grayscale levels converted to mass percentages of calcium ranging from 0 to 38.7% (lower *x*-axis) (**b**). This curve is designated the BMDD and is characterized by the parameters Ca_Width_, Ca_Peak_, F_Peak_, and Ca_Mean_. Only trabecular bones were chosen for the calculation of the BMDD

The PMMA-embedded samples were analyzed using an environmental scanning electron microscope (Quanta 200™, FEI, Hillsboro, OR, USA) coupled with an energy-dispersive X-ray spectrometry detector (QuanTax™, Bruker). The parameters used included an accelerating voltage of 20 kV and a working distance of 10 mm. The bone mineral density distribution (BMDD) was evaluated using the method described by Roschger [15] and adapted by Olejnik [16]. The recorded images (Fig. 1a) are backscattered electron (BSE) images. The BSE images are displayed in grayscale. The BSE grayscale was calibrated using the “atomic number (*Z*) contrast" of reference materials, using PMMA resin (*Z* = 6), aluminum (Al, *Z* = 13), magnesium fluoride (MgF^2^, Zmean = 10), and tricalcium phosphate (β-TCP, Zmean = 14.4). The experimental gray-levels of PMMA and β-TCP were taken as 0 and 38.7% of weight of calcium, respectively. The BE gray-level was converted into weight concentration calcium. The intensity of the grayscale levels is dependent on the amount of calcium. The grayscale curve was extracted from the BSE images (Fig. 1b—upper *x*-axis). This curve corresponded to the BMDD. Through instrument calibration, the grayscale curve was converted into the mass percentage of calcium (Fig. 1b—bottom *x*-axis). The BMDD depicts the percentage of bone surface as a function of the percentage of calcium. Four parameters were calculated from the BMDD: (a) the full-width at half maximum (Ca_Width_), expressed as the mass percentage of calcium; (b) the most frequently occurring calcium concentration (Ca_Peak_), expressed as the weight percentage of calcium; (c) the bone surface frequency of Ca_peak_ (F_Peak_), expressed as a percentage; and d) the average calcium concentration (Ca_Mean_), expressed as the mass percentage of calcium. Calculations were performed using MATLAB software (R2023a; MathWorks, Natick, MA, USA).

BSE images enable the localization of bone surface areas based on their calcium content. The images were segmented into three natures of packets: PMMA resin (black), newly formed bone (new bone), and mature bone (old bone). New and old bone packets were differentiated according to the calcium content: new bone was represented by a calcium content ranging from 0 to 90% of the maximum (primary mineralization), while old bone was represented by a calcium content ranging from 90 to 100% (secondary mineralization) [17]. Standardizing the areas of new bone and old bone based on calcium content allows for the identification of relevant analysis regions for Raman microspectroscopy measurements. Only BSE images containing sufficient trabecular bone were used for this analysis. Cortical bone was not considered in this study.

### Raman Microspectroscopy

**Fig. 2:**
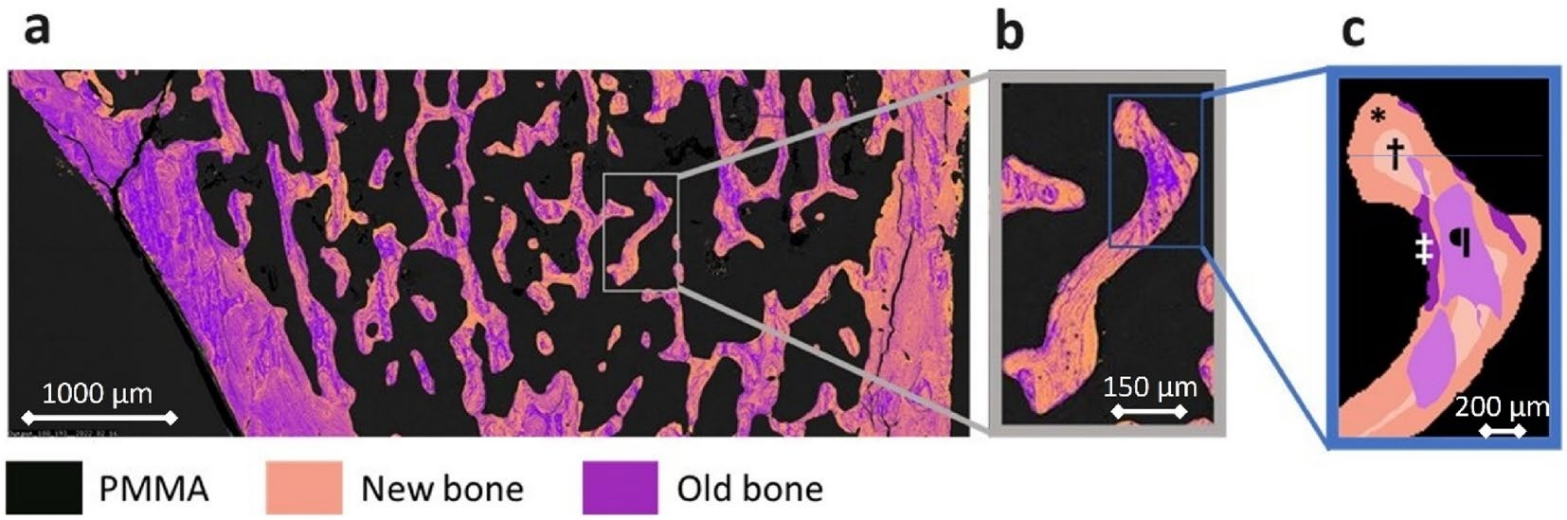
The grayscale BSE image was colored into black (grayscale 0–99), pink (grayscale 100–193, accounting for 90% of mineralization), and purple (grayscale 194–255) to locate new bone and old bone prior to the area selection on the Raman microspectrometer (**a**). A magnified view of a trabecula (**b**). Simplified image of the four categories, showing position (surface or core) and mineralization (new bone or old bone) (**c**). * new bone–trabecular surface; † new bone–trabecular core; ‡ old bone, trabecular surface; ¶ old bone, trabecular core

Raman analyses were conducted using a LabRAM HR800 microspectrometer (HORIBA, Jobin Yvon, Villeneuve d’Ascq, France) equipped with a diode laser (785 nm, 100 mW), a CCD detector (1024 by 256 pixels), and a × 100 objective (NA = 0.80; Olympus, France). The spectral acquisition window was set between 300 and 1800 cm^–1^. The lateral resolution was 1 µm. A scrambler was used to minimize polarization effects. The acquisition time consisted of two acquisitions of 30 s each. Four bone areas were identified from the BSE images (see Fig. 2): new bone–trabecular surface (NB-Tb.S), new bone–trabecular core (NB-Tb.C), old bone–trabecular surface (OB-Tb.S), and old bone–trabecular core (OB-Tb.C). For each sample and each bone area, ten Raman spectra were acquired. All Raman spectra were processed using LabSpec 6 software (HORIBA, Jobin Yvon, France). The physicochemical parameters were calculated using MATLAB software (R2023a; MathWorks, USA) as follows.

The mineral-to-matrix ratio (MMR) describes the amount of mineral relative to the amount of organic matrix in bone [13]. It is the ratio of the area under the ν_1_PO_4_ peak (range 900–990 cm^–1^) to that of the* δ*(CH_2_) peak of collagen (range 1434–1490 cm^–1^). Crystallinity (CRYST) refers to the size and perfection of mineral crystals, which gradually increase with the formation of apatite [13]. This peak is the inverse of the full-width at half maximum of the ν_1_PO_4_ peak. The relative content of carbonate B (CARB) measures the amount of type B carbonates in the bone mineral [13]. It is the ratio of the area under the CO_3_^2−^ type B peak (range 1052–1092 cm^–1^) to that of the ν_1_PO_4_ peak. The hydroxyproline/proline ratio (HPP) provides information about the posttranslational modifications of collagen [18]. It is calculated by the ratio of the intensity of the proline peak (range 828–898 cm^–1^) to that of the hydroxyproline peak (range 828–898 cm^–1^). Collagen maturity (COLL) corresponds to collagen crosslinking modifications [13, 19]. It is calculated by the ratio of the intensity between two successive amide I peaks (1660 and 1690 cm^–1^). The relative content of glycosaminoglycans (GAGs) is indicative of noncollagenous organic constituents [13]. Glycosaminoglycans play a role in assembling the organic content, modulating mineralization and remodeling, and preserving the nonmineralized organic matrix of osteocytes and canaliculi. It is calculated by the ratio of the area under the glycosaminoglycan peak (range 1365–1390 cm^–1^) to that of the amide III peak (range 1243–1269 cm^–1^). These six parameters were measured at four different bone locations: NB-Tb.S, OB-Tb.C, NB-Tb.C, and OB-Tb.S (Fig. 2).

### Statistical Analysis

Qualitative variables are described in terms of frequencies and percentages. Quantitative variables are described in terms of the mean and standard deviation. The normality of distributions was assessed visually and using the Shapiro‒Wilk test.

Comparisons between two elements were conducted using the Student’s *t* test for normally distributed data and the Mann‒Whitney test for nonnormally distributed data. Multiple comparisons were performed using one-way ANOVA for normally distributed data and the Kruskal‒Wallis test for nonnormally distributed data. When a significant difference was found, a post hoc Dunn’s test was applied to characterize the difference. The relationship between the parameters and adiposity was assessed using the Pearson correlation coefficient and its associated test. Each correlation was assessed for the whole sample and for separate groups. For parameters significantly associated with adiposity at a threshold of 0.10, we investigated, using the same method, the relationship between the parameter and adiposity in each of the three compartments (D1, D2, and D3). Statistical comparison analyses were conducted using GraphPad Prism v7.0 software (GraphPad Software, San Diego, CA, USA). Statistical correlation analyses were performed using SAS software (SAS Institute version 9.4). The significance level was set at 5%.

## Results

### Effects of Ovariectomy on Trabecular Bone

#### Bone Loss and BMAT Gain

**Fig. 3:**
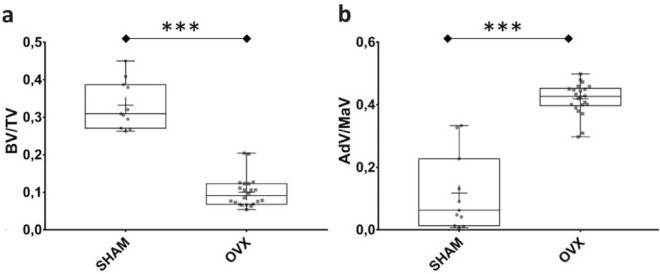
Bone volume/total volume (BV/TV) ratios of the SHAM and OVX groups in trabecular bone of the tibiae. The region of interest is a cross-sectional slice of 2 mm thickness, located 1.5 mm below the growth plate. **a** The adipose volume-to-marrow volume (AdV/MaV) ratios of the SHAM and OVX groups (**b**). The cross represents the mean. ***p < 0.001

The BV/TV and the AdV/MaV within the trabecular bone were compared between the SHAM and OVX groups. The OVX group exhibited a significantly lower BV/TV ratio than did the SHAM group (median = 11.1% [Q1 = 7.5%, Q3 = 12.4%] vs. median = 31.0% [Q1 = 28.3%, Q3 = 38.6%], *p* < 0.001—Fig. 3a). The OVX group exhibited a significantly greater AdV/MaV ratio than did the SHAM group (median = 42.7% [Q1 = 36.7%, Q3 = 48.3%] vs. median = 7.5% [Q1 = 1.2%, Q3 = 23.1%], *p* < 0.001—Fig. 3b).

**Fig. 4:**
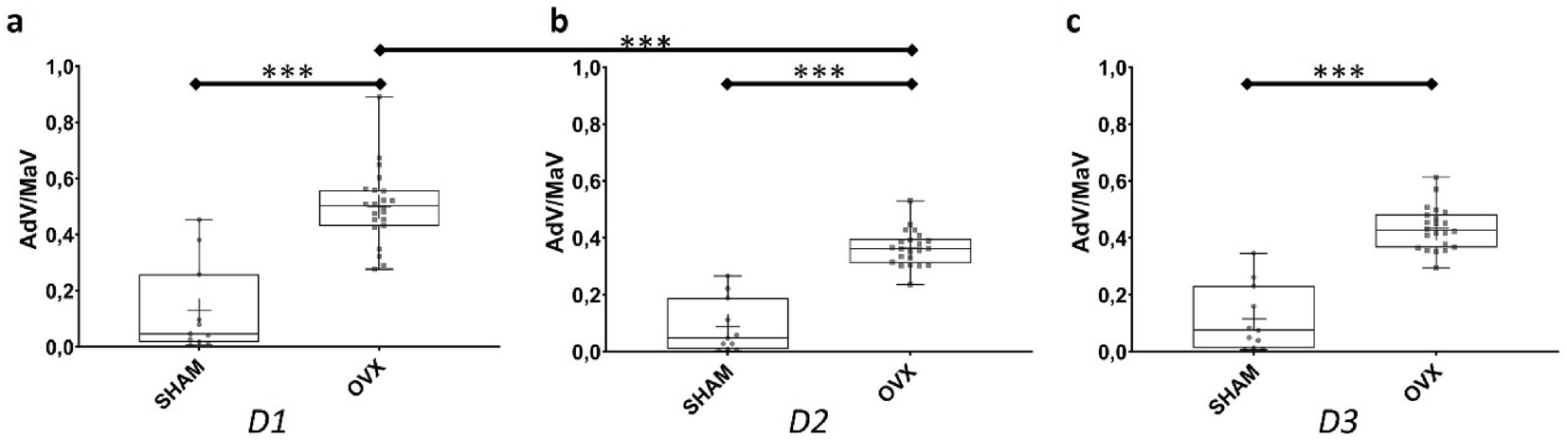
Adipose volume-to-total volume (AdV/MaV) ratios of the SHAM and OVX groups based on spatial distribution. **a** D1: 0–20 µm; **b** D2: 20–40 µm; **c** D3: 40–60 µm. The cross represents the mean. ***p < 0.001

The AdV/MaV parameter was assessed based on its proximity to the trabecular bone surface. The bone marrow adjacent to the bone surface was segmented into three compartments according to their distance from the surface: D1 (0–20 µm), D2 (20–40 µm), and D3 (40–60 µm). The difference in the AdV/MaV ratios between the SHAM and OVX groups remained significant in compartments D1, D2, and D3 (*p* < 0.001 for all three—Fig. 4). The parameter AdV/MaV did not differ across the three compartments in the SHAM group (D1–D2: *p* = 0.73; D2−D3: p = 0.87; D1−D3: *p* = 0.97—Fig. 4). In the OVX group, the AdV/MaV ratio was greater at D1 than at D2 (*p* < 0.001). The other compartments did not show differences (D2−D3: *p* = 0.05; D1−D3: *p* = 0.07).

#### Bone Mineral Density Distribution (BMDD)

**Fig. 5:**
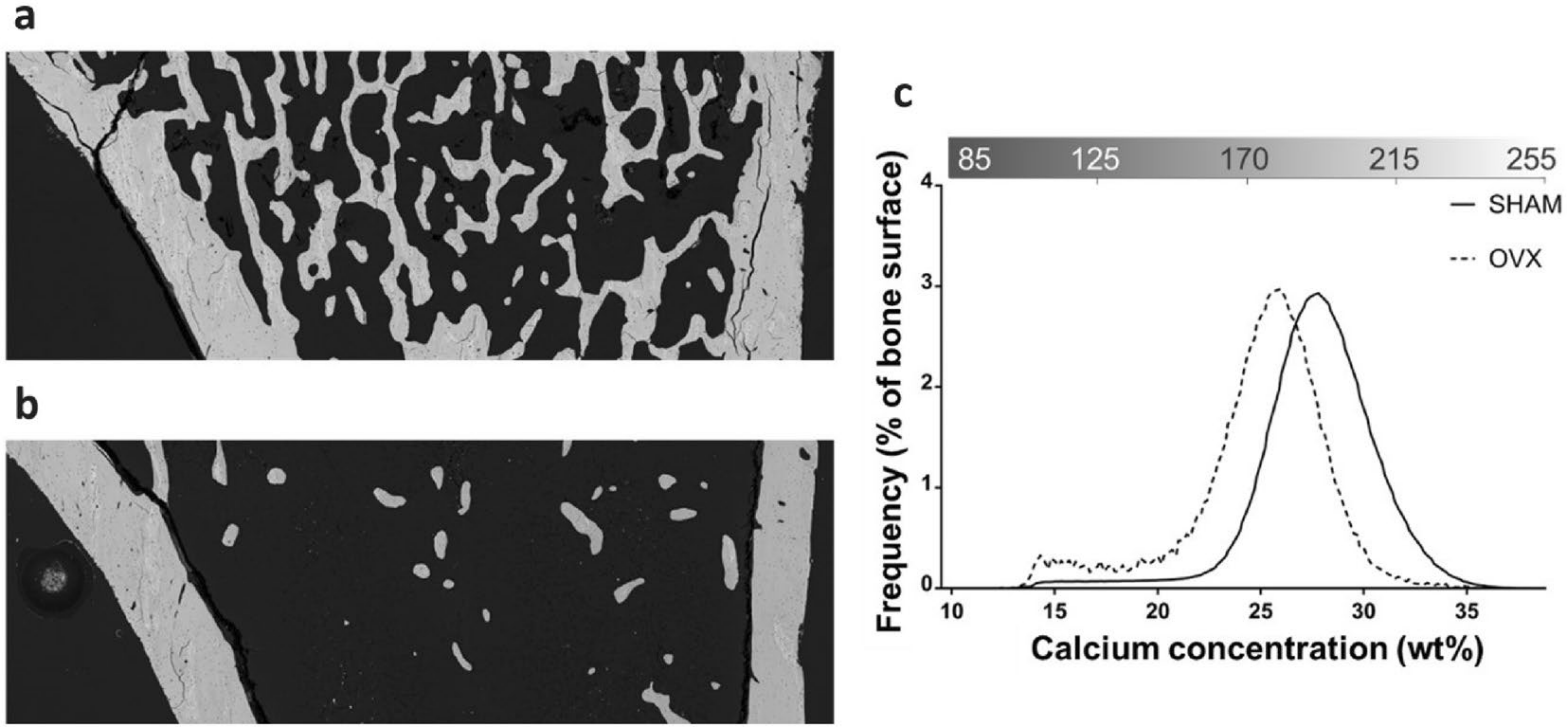
Backscattered electron (BSE) image of a proximal metaphysis of a tibia from the SHAM group (**a**). BSE image of a proximal metaphysis of a tibia from the OVX group (**b**). BMDD of the SHAM (solid lines) and OVX (dash lines) groups (**c**)

The trabecular bones of the SHAM and OVX groups were analyzed using ESEM imaging and qBEI. Figure 5a and b shows a representative calcium content representation on BSE images of the trabecular bones of the SHAM and OVX groups, respectively. The SHAM trabecular bones exhibited quantitative modifications, with a higher number of trabeculae compared to those in the OVX group. The trabecular BMDD of the OVX group showed a significant shift toward a low concentration of Ca compared to the BMDD of the SHAM group (Fig. 5c, Table 1). The Ca_Width_ of the OVX group was significantly lower compared to the Ca_Width_ of the SHAM group (median = 4.01 [Q1 = 3.89, Q3 = 4.27] vs. median = 4.56 [Q1 = 4.28, Q3 = 5.03], *p* < 0.001). The Ca_Mean_ of the OVX group was significantly lower compared to the Ca_Mean_ of the SHAM group (median = 25.33% [Q1 = 24.90%, Q3 = 25.66% vs. median = 27.59% [Q1 = 27.24%, Q3 = 28.60], *p* < 0.001).

**Table 1:** Overview of the parameters measured in the two groups SHAM and OVX

		SHAM		OVX		*p*
		*N*	Mean (SD) Median (Q1;Q3)	*N*	Mean (SD) Median (Q1;Q3)	
*µCT*						
**BV/TV (%)**		**11**	**31.0 (28.3;38.6)**	**22**	**11.1 (7.5;12.4)**	** < 0.001**^**a**^
**AdV/MaV (%)**		**11**	**6.2 (1.3;22.7)**	**22**	**42.7 (39.7;45.2)**	** < 0.001**^**a**^
**AdV/MaV**_**D1**_** (%)**		**11**	**4.6 (1.7;25.8)**	**22**	**50.3 (43.1;55.9)**	** < 0.001**^**a**^
**AdV/MaV**_**D2**_** (%)**		**11**	**4.8 (0.9;18.8)**	**22**	**36.2 (31.2;39.7)**	** < 0.001**^**a**^
**AdV/MaV**_**D3**_** (%)**		**11**	**7.5 (1.2;23.1)**	**22**	**42.7 (36.7;48.3)**	** < 0.001**^**a**^
*BMDD*						
**Ca**_**Width**_** (wt%)**		**11**	**4.56 (4.28;5.03)**	**21**	**4.01 (3.89;4.27)**	** < 0.001**^**a**^
**Ca**_**Peak**_** (wt%)**		**11**	**27.76 (27.41;28.62)**	**21**	**25.69 (25.34;26.38)**	** < 0.001**^**a**^
**Ca**_**Mean**_** (wt%)**		**11**	**27.59 (27.24;28.60)**	**21**	**25.33 (24.90;25.66)**	** < 0.001**^**a**^
**F**_**Peak**_** (%)**		**11**	**3.234 (0.297)**	**21**	**3.556 (0.257)**	**0.003**^**b**^
*Physico−chemical parameters: bone ultrastructural composition (Raman)*						
*NB-Tb.S*	**MMR_NB-Tb.S**	**11**	**11.95 (1.836)**	**20**	**10.63 (1.401)**	**0.03**^**b**^
	CARB_NB-Tb.S	11	0.094 (0.008)	20	0.097 (0.008)	0.46^**b**^
	**CRYST_NB-Tb.S**	**11**	**0.056 (0.001)**	**20**	**0.057 (0.001)**	**0.003**^**b**^
	COLL_NB-Tb.S	11	1.427 (0.087)	20	1.534 (0.182)	0.08^**b**^
	HPP_NB-Tb.S	11	0.530 (0.480;0.570)	20	0.540 (0.503;0.603)	0.36^**a**^
	GAG_NB-Tb.S	11	0.063 (0.022)	20	0.053 (0.013)	0.11^**b**^
*OB-Tb.C*	MMR_OB-Tb.C	11	16.65 (2.631)	7	14.97 (3.369)	0.25^**b**^
	CARB_OB-Tb.C	11	0.103 (0.012)	7	0.107 (0.014)	0.56^**b**^
	CRYST_OB-Tb.C	11	0.056 (0.001)	7	0.056 (0.000)	0.61^**b**^
	COLL_OB-Tb.C	11	1.380 (0.159)	7	1.479 (0.079)	0.15^**b**^
	HPP_OB-Tb.C	11	0.419 (0.067)	7	0.477 (0.091)	0.14^**b**^
	GAG_OB-Tb.C	11	0.080 (0.029)	7	0.085 (0.034)	0.75^**b**^
*NB-Tb.C*	**MMR_NB-Tb.C**	**11**	**14.23 (1.602)**	**19**	**11.71 (1.497)**	** < 0.001**^**b**^
	CARB_NB-Tb.C	11	0.102 (0.005)	19	0.106 (0.007)	0.13^**b**^
	**CRYST_NB-Tb.C**	**11**	**0.056 (0.000)**	**19**	**0.057 (0.001)**	** < 0.001**^**b**^
	COLL_NB-Tb.C	11	1.488 (0.093)	19	1.434 (0.108)	0.18^**b**^
	**HPP_NB-Tb.C**	**11**	**0.496 (0.056)**	**19**	**0.552 (0.073)**	**0.04**^**b**^
	GAG_NB-Tb.C	11	0.053 (0.019)	18	0.057 (0.011)	0.45^b^
*OB-Tb.S*	MMR_OB-Tb.S	11	18.30 (4.088)	3	12.68 (4.364)	NA
	CARB_OB-Tb.S	11	0.109 (0.010)	3	0.114 (0.014)	NA
	CRYST_OB-Tb.S	11	0.056 (0.001)	3	0.057 (0.000)	NA
	COLL_OB-Tb.S	11	1.613 (0.246)	3	1.474 (0.122)	NA
	HPP_OB-Tb.S	11	0.401 (0.078)	3	0.410 (0.044)	NA
	GAG_OB-Tb.S	8	0.075 (0.026)	3	0.063 (0.035)	NA

*BMAT* bone marrow adipose tissue, *µCT* microcomputed tomography; *BMDD* bone mineral density distribution; *AdV/MaV* adipose volume-to-marrow volume ratio; *BV/TV* bone volume-to-total volume ratio; *Ca*_*Width*_ full-width at half maximum of the calcium distribution; *Ca*_*Peak*_ most frequently occurring calcium concentration; *F*_*Peak*_ bone surface frequency of the most frequently occurring calcium concentration; *Ca*_*Mean*_ average calcium concentration;* MMR* mineral-to-matrix ratio; *CARB* relative carbonate B content; *CRYST* mineral crystals’ crystallinity; *COLL* collagen maturity; *HPP* hydroxyproline/proline ratio; *GAG* glycosaminoglycans; *NB-Tb.S* new bone–trabecular surface; *OB-Tb.C* old bone–trabecular core; *NB-Tb.C* new bone–trabecular core; *OB-Tb.S* old bone–trabecular surface; *N* sample size; *NA* not applicable; Bold indicates significant differences at the *p* = 0.05 threshold

^a^Mann‒Whitney test; ^b^Student’s test

One left tibia sample did not contain trabecular bone; the corresponding mineral and bone composition parameters were not evaluated.

#### Raman Microspectroscopy

Raman spectra were acquired in bone packets depending on bone age. The bone packets were identified in BSE images and labeled as follows: NB-Tb.C, NB-Tb.S, OB-Tb.C, and OB-Tb.S. The physicochemical parameters were calculated and compared between the SHAM and OVX groups depending on the bone age.

The OVX group exhibited a lower MMR than the SHAM group in the NB-Tb.S (10.63 ± 1.4 vs. 11.95 ± 1.8, *p* = 0.03) and in the NB-Tb.C (11.71 ± 1.50 vs. 14.23 ± 1.60, *p* < 0.001). The OVX group exhibited a higher CRYST than the SHAM group in the NB-Tb.S (0.057 ± 0.001 vs. 0.056 ± 0.000, *p* = 0.003) and the NB-Tb.C (0.057 ± 0.001 vs. 0.056 ± 0.001, *p* < 0.001). We did not find significant differences in the parameters of bone composition in the old bone (surface or core). No significant difference was found between the OVX and SHAM groups for the following parameters: CARB, COLL, and GAG. Due to the low number of trabeculae in OVX (Fig. 5b), some samples did not have old bone (according to Ruffoni description). Thus, some Raman acquisitions could not be performed on old bone in these specific samples.

### Correlations with BMAT

Correlations were investigated to explore potential associations between BMAT and bone quality. Correlations were assessed for pooled data (SHAM and OVX) and for each group. The correlations between BMAT and bone loss, mineral parameters, and bone composition are provided in Table 2.

**Table 2:** Correlations of the AdV/MaV ratios of the whole sample and separate groups

		Whole sample			SHAM			OVX		
		*N*	*r*	*p*	*N*	*r*	*p*	*N*	*r*	*p*
*Correlation with bone density*										
**$ BV/TV**		**33**	**–0.79**	** < 0.001**	11	0.41	0.21	**22**	**–0.54**	**0.009**
*Correlations with bone mineral density distribution*										
**$ Ca**_**Width**_		**32**	**–0.59**	** < 0.001**	11	–0.06	0.87	21	–0.27	0.24
**$ Ca**_**Peak**_		**32**	**–0.67**	** < 0.001**	11	–0.16	0.65	21	–0.10	0.67
**$ F**_**Peak**_		**32**	**0.48**	**0.005**	11	0.04	0.91	21	0.19	0.41
**$ Ca**_**Mean**_		**32**	**–0.75**	** < 0.001**	11	–0.20	0.57	21	–0.22	0.35
*Correlations with Raman microspectroscopy*										
*NB-Tb.S*	$ MMR_NB-Tb.S	31	–0.37	0.04	11	–0.15	0.67	20	0.05	0.85
	$ CARB_NB-Tb.S	31	0.04	0.85	11	–0.30	0.39	20	–0.05	0.85
	**$ CRYST_NB-Tb.S**	**31**	**0.55**	**0.001**	11	0.38	0.26	20	0.17	0.49
	$ COLL_NB-Tb.S	31	0.26	0.15	11	–0.02	0.96	20	–0.06	0.82
	$ HPP_NB-Tb.S	31	–0.01	0.94	11	–0.10	0.79	20	0.01	0.98
	$ GAG_NB-Tb.S	31	–0.20	0.28	11	0.10	0.77	20	0.18	0.46
*OB-Tb.C*	$ MMR_OB-Tb.C	18	–0.22	0.39	11	0.19	0.59	7	–0.41	0.38
	$ CARB_OB-Tb.C	18	0.10	0.69	11	0.01	0.97	7	–0.15	0.76
	$ CRYST_OB-Tb.C	18	0.21	0.41	11	0.15	0.67	7	0.35	0.46
	$ COLL_OB-Tb.C	18	0.09	0.74	11	–0.34	0.32	7	–0.48	0.30
	$ HPP_OB-Tb.C	18	0.35	0.15	11	0.02	0.96	7	0.37	0.44
	$ GAG_OB-Tb.C	18	0.13	0.60	11	0.31	0.37	7	–0.36	0.45
*NB-Tb.C*	**$ MMR_NB-Tb.C**	**30**	**–0.65**	** < 0.001**	11	–0.33	0.33	19	–0.22	0.38
	$ CARB_NB-Tb.C	30	0.20	0.29	11	0.07	0.84	19	–0.28	0.26
	$ CRYST_NB-Tb.C^*^	**30**	**0.49**	**0.006**	11	–0.33	0.34	19	0.08	0.76
	$ COLL_NB-Tb.C	30	–0.22	0.24	**11**	**0.63**	**0.03**	**19**	**–0.69**	** < 0.001**
	$ HPP_NB-Tb.C	30	0.39	0.03	11	0.01	0.98	19	0.30	0.21
	$ GAG_NB-Tb.C	29	0.17	0.39	11	0.18	0.60	18	–0.16	0.54
*OB-Tb.S*	$ MMR_OB-Tb.S	14	–0.31	0.28	11	0.01	0.98	3	NA	NA
	$ CARB_OB-Tb.S	14	0.31	0.30	11	0.10	0.78	3	NA	NA
	$ CRYST_OB-Tb.S	14	0.18	0.56	11	0.10	0.78	3	NA	NA
	$ COLL_OB-Tb.S	14	–0.07	0.82	11	0.25	0.48	3	NA	NA
	$ HPP_OB-Tb.S	14	–0.20	0.49	11	–0.37	0.27	3	NA	NA
	$ GAG_OB-Tb.S	11	–0.17	0.64	8	–0.06	0.89	3	NA	NA

*AdV/MaV* adipose volume-to-marrow volume ratio; *BV/TV* bone volume--to-total volume ratio; *Ca*_*Width*_ full-width at half maximum of the calcium distribution; *Ca*_*Peak*_ most frequently occurring calcium concentration; *F*_*Peak*_ bone surface frequency of the most frequently occurring calcium concentration; *Ca*_*Mean*_ average calcium concentration; *MMR* mineral-to-matrix ratio; *CARB* relative carbonate B content; *CRYST* mineral crystals’ crystallinity; *COLL* collagen maturity; *HPP* hydroxyproline/proline ratio; *GAG* glycosaminoglycans; *NB-Tb.S* new bone–trabecular surface; *OB-Tb.C* old bone–trabecular core; *NB-Tb.C* new bone–trabecular core; *OB-Tb.S* old bone–trabecular surface; *N* sample size. The values are expressed as the Pearson correlation coefficient (*r*) and significance (*p*). The symbol $ indicates correlation with the AdV/MaV ratio. Strong correlations (*r* > 0.50) that are statistically significant at the *p* = 0.05 threshold are highlighted in bold. *CRYST_NB-Tb.C (*r* = 0.49) has been included in the strong correlations

When data are pooled, a strong negative correlation was observed between the AdV/MaV ratio and the BV/TV ratio (*r* =  − 0.79, *p* < 0.001). Strong correlations between the AdV/MaV ratio and mineral parameters were also observed (*r* =  − 0.59, *p* < 0.001 Ca_Width_; *r* =  − 0.67, *p* < 0.001 with Ca_Peak_; *r* = 0.48, *p* = 0.005; and *r* =  − 0.75, *p* < 0.001 with Ca_Mean_). Three strong correlations between the AdV/MaV ratio and Raman parameters were observed: CRYST_NB-Tb.S, MMR_NB-Tb.C, and CRYST_NB-Tb.C (*r* = 0.55, *p* = 0.001; *r* =  − 0.65, *p* < 0.001; and *r* = 0.49, *p* = 0.006, respectively).

The correlation between the AdV/MaV ratio and the BV/TV ratio is not significant in SHAM, and is strong and negative in OVX (*r* =  − 0.54, *p* = 0.009). The correlation between the AdV/MaV ratio and COLL_NB-Tb.C is strong and positive in SHAM (*r* = 0.63, *p* = 0.03), and strong and negative in OVX (*r* =  − 0.69, *p* < 0.001).

The analysis did not reveal a correlation between the AdV/MaV ratio and CARB or GAG (across all zones). In the old bone–trabecular cores, no correlations were observed either.

### Correlation with BMAT Based on Spatial Distribution

In the previous section, eight parameters were significantly correlated (*p* < 0.05) when evaluating the pooled SHAM and OVX groups. These eight parameters were analyzed based on their spatial distribution (D1, D2, and D3). The correlations between BMAT and eight parameters are presented in Table 3 depending on their spatial distribution. The direction of the correlations was consistent across compartments. However, we noticed slight differences according to the spatial distribution for AdV/MaV $ BV/TV, AdV/MaV $ F_Peak_, AdV/MaV $ MMR_NB-Tb.S, and AdV/MaV $ HPP_NB-Tb.C, but the correlations remain on the same order of magnitude.

**Table 3:** Pearson correlations of the pooled parameters AdV/MaV_D1_, AdV/MaV_D2_, and AdV/MaV_D3_ (SHAM and OVX) based on the spatial distribution of significantly correlated parameters (*p* < 0.05)

	$ AdV/MaV_D1_			$ AdV/MaV_D2_			$ AdV/MaV_D3_		
	*N*	*r*	*p*	*N*	*r*	*p*	*N*	*r*	*p*
BV/TV	33	–0.70	< 0.001	33	–0.81	< 0.001	33	–0.80	< 0.001
Ca_Width_	32	–0.60	< 0.001	32	–0.61	< 0.001	32	–0.58	< 0.001
Ca_Peak_	32	–0.70	< 0.001	32	–0.69	< 0.001	32	–0.68	< 0.001
F_Peak_	32	0.57	< 0.001	32	0.44	0.01	32	0.41	0.02
Ca_Mean_	32	–0.75	< 0.001	32	–0.78	< 0.001	32	–0.77	< 0.001
MMR_NB-Tb.S	31	–0.46	0.009	31	–0.37	0.04	31	–0.35	0.05
CRYST_NB-Tb.S	31	0.54	0.002	31	0.51	0.003	31	0.50	0.003
MMR_NB-Tb.C	30	–0.67	< 0.001	30	–0.65	< 0.001	30	–0.63	< 0.001
CRYST_NB-Tb.C	30	0.43	0.02	30	0.47	0.008	30	0.48	0.007
HPP_NB-Tb.C	30	0.33	0.08	30	0.42	0.02	30	0.40	0.03

*AdV/MaV* adipose volume-to-marrow volume ratio; *BV/TV* bone volume-to-total volume ratio; *Ca*_*Width*_ full-width at half maximum of the calcium distribution; *Ca*_*Peak*_ most frequently occurring calcium concentration; *F*_*Peak*_ bone surface frequency of the most frequently occurring calcium concentration; *Ca*_*Mean*_ average calcium concentration; *MMR* mineral-to-matrix ratio; *CARB* relative carbonate B content; *CRYST* mineral crystals’ crystallinity; *COLL* collagen maturity; *HPP* hydroxyproline/proline ratio; *GAG* glycosaminoglycans; *NB-Tb.S* new bone–trabecular surface; *NB-Tb.C* new bone–trabecular core; $ indicates the correlation. *N* = sample size. The values are expressed as the Pearson correlation coefficient (*r*) and significance (*p*)

## Discussion

The main objective was to evaluate the spatial correlation between BMAT and bone quality in settings of OVX. Secondary objectives included describing the long-term effects of ovariectomy on bone.

### The Trabecular Bone in the OVX Model Exhibits Characteristics of Postmenopausal Osteoporosis in Terms of Bone Loss and Composition

Liu et al. reported a 73% decrease in proximal tibia density in 15-month-old ovariectomized (OVX) rats [20]. Our study found a 70% lower BV/TV ratio in OVX rats compared to controls, aligning with existing literature. BMAT significantly increased (+ 257%) in OVX rats. Limited animal model literature exists on BMAT, but in humans, Li et al. observed greater BMAT in osteoporotic women (+ 7%, *n* = 51) [21], with Sheu and Cauley noting BMAT values from 8 to 45%. Our results indicated a more pronounced difference in rats after 9 months post-ovariectomy. Coutel et al*.* introduced analysis of BMAT distribution, showing a significant increase in adipocytes (AdV/MaV) near the bone surface in OVX rats (*p* < 0.001) [8], consistent with our results.

Regarding bone mineralization, Brennan et al. found a 5% lower mean calcium content (Ca_Mean_) in the proximal femur of OVX sheep [22]. We observed a 7% reduction in Ca_Mean_ in OVX rats, confirming the long-term effects of ovariectomy. Valenta noted no significant Ca_Mean_ difference but a 14% reduction in full-width at half maximum (Ca_Width_) in OVX rats, suggesting long-term impacts on bone [23].

The MMR and the CARB reflect bone quality. Models have shown decreased MMR and increased CARB in osteoporosis [24, 25]. Our study confirmed lower MMR (− 7%) and a non-significant 4% increase in CARB in OVX rats, indicating reduced bone quality. Paschalis et al*.* found higher COLL and CRYST in osteoporotic women, with lower GAG [26]. We observed higher CRYST (+ 1%) in OVX rats, but no significant changes in COLL or GAG. HPP, linked to collagen modifications, was not significantly altered, though previous studies reported increases [27]. We found a 7% increase in HPP in OVX rats. The observed increase in CRYST between the SHAM and OVX groups, though relatively small, falls within the range of previous studies and may suggest a subtle biological relationship, especially given the sensitivity of our measurements at the micrometer scale.

Among 22 OVX samples, only 7 contained old bone in the trabecular core (OB-Tb.C) and 3 contained old bone on the trabecular surface (OB-Tb.S). Twelve OVX sample did not have OB-Tb.C and OB-Tb.S bone packets for Raman analysis. This suggests less trabecular bone surface (see Fig. 5b) and mature bone in osteoporosis, complicating analysis in small species like rodents. Our OVX model results align with existing literature on bone quality and BMAT [28], validating its use in studying postmenopausal osteoporosis.

### BMAT is Correlated with the Mineral Component of Trabecular Bone Quality

BMAT is negatively correlated with volumetric bone mineral density (vBMD) in postmenopausal osteoporotic women [29]. BMAT, a unique fat reservoir within bone cavities, is associated with obesity, which in turn is positively correlated with bone mineral density (BMD) [30]. However, fracture risk is higher in obese individuals, potentially due in part to BMAT's negative impact on bone quality and quantity [31]. Our study revealed a negative correlation between BMAT and bone microarchitecture.

Schwartz et al. (2013) also reported this negative correlation between BMAT and vBMD in postmenopausal women [29]. However, research on BMAT's interaction with bone quality at the trabecular level is limited. Our study aimed to fill this gap. We found that the AdV/MaV ratio correlated with bone mineral density distribution (BMDD) parameters. Increased BMAT was linked to decreases in Ca_Peak_, Ca_Mean_, and Ca_Widt_h, and an increase in F_Peak_, indicating a shift toward less mineralized and potentially weaker bone. This correlation was significant in new bone, suggesting a disrupted bone remodeling.

Due to limited correlations observed within the OVX group, our subsequent analysis and discussion focused on the entire sample. We carefully checked the data for consistency to ensure a cautious interpretation of our findings.

CRYST was positively associated with BMAT in new bone of pooled data over both groups (SHAM and OVX), suggesting adipocytes could influence apatite crystal maturation. This suggests that adipocytes and their secreted products may influence apatite crystal maturation through lipid metabolism's effect on mineralization, secretion of bone-modulating factors, and alteration of bone remodeling dynamics [32, 33]. No significant correlation was found in old bone, likely due to substantial bone loss and increased remodeling rates, reducing old bone amount in OVX samples.

Our study showed a decrease in old bone surface ratio from 47% in the SHAM group to 13% in the OVX group. While we quantified this reduction, we previously also noted increased BMAT in an OVX context [8]. The degree of unsaturation of fatty acids in BMAT affects its lipotoxic impact on bone quality [33].

In this study, increased BMAT correlated with altered mineral content, decreased CaPeak and CaMean in trabecular bone, and decreased MMR in new bone. Although the direct impact of adipocytes on bone quality is underexplored, recent evidence presents conflicting views. Entz et al. highlighted variations in BMAT's extracellular matrix, suggesting an influence on bone mineral quality [34], while other research offers counterarguments that moderate this perspective [35].

### Limitations

The main objective was to evaluate the spatial relationship between BMAT and bone quality in settings of OVX. Our results were in agreement with previous studies confirming the biological response of the animals to the surgery. Despite the coherence of our results, the study has some limitations. As animal model, it has its own obvious limits, as it cannot truly reproduce the human osteoporosis (e.g., no fragility fracture in the animal model).

Considering the analytical techniques, outcomes from Raman, qBEI, and µCT are surrogates to evaluate the mineral content and bone marrow, and should be taken with caution. The relative bone tissue age was approximated by qBEI which is not the gold standard method and may induce some variability in the Raman outcomes. The use of fluorescent label would improve the evaluation of tissue age. However, our Raman results were found in agreement with previous studies. It supports that our approximation of tissue age was done correctly.

Our results concerning bone marrow are mainly representative of the regulated bone marrow adipose tissue (rBMAT) and not constitutive BMAT due to the selection of the proximal tibial red marrow as an analysis region.

The lack of significant correlations in our study likely reflects limitations such as small sample size, data variability, and uncontrolled confounding factors, rather than an absence of a true relationship between BMAT and bone quality. In complex systems like the OVX/osteoporosis model, correlations may not always be evident, underscoring the need for further research with larger, more controlled studies.

## Conclusion

In conclusion, we investigated bone quality at various scales, including mineral bone density distribution and bone ultrastructural composition, in relation to BMAT changes in the proximal metaphysis of tibiae in setting OVX. Our findings were in agreement with previous studies, such as reduced microarchitecture and mineral content, structural alterations in the mineral component, and increased bone marrow adipose tissue. Additionally, we examined the correlations between BMAT and bone quality in setting OVX, considering the spatial distribution of adipocytes. Interestingly, BMAT was found to be associated with parameters of the mineral component, including bone mineral density distribution, the mineral-to-matrix ratio, and hydroxyapatite crystal perfection. As BMAT increased, these parameters shifted toward an impaired bone quality, especially near the trabecular newly formed bone areas, as identified by the qBEI. These findings provide new insights for further investigations into the relationship between BMAT and bone tissue during postmenopausal osteoporosis.

## Data Availability

The data that support the findings of this study are available from the corresponding author, Maxime Bedez, upon reasonable request.

## Abbreviations

µCT: Microcomputed tomography
AdV/MaV: Adipose volume-to-marrow volume ratio
BMAd: Bone marrow adipocyte
BMAT: Bone marrow adipose tissue
BMD: Bone mineral density
BMDD: Bone mineralization density distribution
BSE image: Backscattered electron image
BV/TV: Bone volume-to-total volume ratio
Ca_Mean_: Average calcium concentration
Ca_Peak_: Most frequently occurring calcium concentration
CARB: Relative carbonate B content
Ca_Width_: Full-width at half maximum of the calcium distribution
COLL: Collagen maturity
CRYST: Mineral crystals’ crystallinity
ESEM: Environmental scanning electron microscopy
F_Peak_: Bone surface frequency of the most frequently occurring calcium concentration
HPP: Hydroxyproline/proline ratio
MgF_2_: Magnesium fluoride
MMR: Mineral-to-matrix ratio
NBF: Neutral buffered formalin
NB-Tb.C: New bone, trabecular core
NB-Tb.S: New bone, trabecular surface
OB-Tb.C: Old bone, trabecular core
OB-Tb.S: Old bone, trabecular surface
OVX: Ovariectomized group
PBS: Phosphate-buffered saline
GAGs: Glycosaminoglycans
PMMA: Polymethylmethacrylate
qBEI: Quantitative backscattered electron imaging
SHAM: Sham-operated group
vBMD: Volumetric bone mineral density
β-TCP: Tricalcium phosphate
